# Editorial: Application of bioinformatics, machine learning, and artificial intelligence to improve diagnosis, prognosis and treatment of cancer

**DOI:** 10.3389/fragi.2025.1763491

**Published:** 2026-01-02

**Authors:** Tinka Vidović, Petar Ozretić

**Affiliations:** 1 Moltech Innovations, Zagreb, Croatia; 2 Laboratory for Hereditary Cancer, Division of Molecular Medicine, Ruđer Bošković Institute, Zagreb, Croatia

**Keywords:** cancer biomarkers, cancer diagnosis and therapy, cancer drug targets, computational cancer biology, computational drug discovery artificial intelligence, bioinformatics, machine learning

In recent years, omics approaches have yielded great advances in cancer research and have provided new in-depth insights into the processes involved in cancer development and progression. Practical use of the information contained within this huge amount of data requires computational approaches such as bioinformatics, machine learning (ML), and artificial intelligence (AI). These computational methods, together with omics data from large databases, such as The Cancer Genome Atlas (TCGA) and Gene Expression Omnibus (GEO), can now be used to develop cancer biomarkers, novel anti-cancer drug targets, and both novel and repurposed treatment options for cancer. Considering the application of versatile computational methods in cancer research, we collected original research articles in this Research Topic to present the novel discovery of potential cancer drug targets, prognostic biomarkers, or therapeutic interventions.

Lower extremity deep vein thrombosis (DVT) is a frequent postoperative complication, occurring in up to 40% of patients with colorectal cancer. Liu et al. used an ML model optimized for predicting an individual’s risk of DVT in colorectal cancer patients. Given the prevalence of DVT and that traditional risk assessments may not be accurate indicators of true risk, they showed that the XGBoost model (Chen and Guestrin, 2016) has strong potential for improving early detection and treatment in clinical settings.

Identifying novel biomarkers for predicting patient survival time is of crucial practical clinical significance, since it could lead to better patient stratification and treatment decisions. Li et al. used the reactive oxygen species (ROS)-related signature genes, which they identified using the TCGA data, to predict the prognosis and chemotherapy response of patients with bladder cancer. They did not only identify 17 ROS-related genes that exhibited good overall survival in bladder cancer patients, but also 11 potential small molecular drugs that target these ROS-related genes using the Connectivity Map (CMap) database (Lamb et al., 2006).

Using ML models to predict response to treatment could lead to the development of more personalized treatment, leading to significant improvement in patient outcomes. Guo et al. developed the Artificial Intelligence-Derived Anoikis Signature (AIDAS), a novel machine learning-based prognostic tool for breast cancer. AIDAS identifies key gene expression patterns related to anoikis, a form of programmed cell death triggered by detachment from the extracellular matrix. Using AIDAS, the authors found they could more accurately predict breast cancer outcomes compared to existing prognostic models. They discovered that patients with low AIDAS levels may be more responsive to immunotherapy, while those with high AIDAS levels are more susceptible to certain chemotherapies like methotrexate.

Zhu et al. showed the crucial role of autophagy in acute myeloid leukemia (AML) prognosis, identifying essential autophagy genes that correlate with patient survival. Using ML, they developed a predictive model that aids risk stratification and suggested potential therapeutic targets. Their findings also reveal a link between autophagy and the immune microenvironment, offering insights for future research and clinical applications.

Due to the large difference in survival of patients with moderately differentiated gastric adenocarcinoma (MDGA) with distant metastases and without metastases, it becomes important to predict the occurrence of distant metastases after surgical treatment, after morphological examination of all removed lymph nodes, and after final staging of the disease. Yang et al. collected data from MDGA patients from the Surveillance, Epidemiology, and End Results (SEER) database from 2010 to 2019, as well as data from MDGA patients in China. Based on these data they conducted univariate and multivariate analyses, and factors were identified that contribute to the occurrence of distant metastases and worsen the prognosis of the disease.

Prostate cancer is a highly metastatic tumor, and it is estimated that about 50% of patients with advanced disease will develop bone metastases. Once bone metastasis occurs, it is incurable and is significantly associated with mortality. The STING signaling pathway is an important transduction mechanism in innate immunity and viral defense, and it has been demonstrated that this pathway plays a key role in tumorigenesis and metastasis (Wang et al., 2025). In their study, Li et al. extrapolated three key STING pathway genes related to bone metastasis based on a machine learning algorithm. After comprehensive analysis, it was verified that these three genes have key roles in prostate cancer development, metastasis, and tumor immunity, while RELA or transcription factor p65 is a highly potential therapeutic target.

Ding et al. explored the role of FAT1, which is crucial for cellular adhesion and cell signaling, in lung cancer cell lines. The authors identified *FAT1* mutations in five out of thirty-seven individuals diagnosed with non-small cell lung cancer (NSCLC), using next-generation sequencing (NGS) technology. These mutations included four missense mutations and one splice variant. The frequency of *FAT1* mutations was the third highest, following those in *EGFR* and *TP53* genes. The study further demonstrated correlations between *FAT1* expression and methylation with the malignancy of certain cancer types. Knockdown of *FAT1* in A549 and H1299 lung cancer cell lines led to downregulated PD-L1 expression. Additionally, *FAT1* knockdown significantly inhibited cell proliferation, colony formation, and migration. It also affected the cell cycle and the FAK-YAP/TAZ signaling pathway, ultimately inhibiting the proliferation of lung cancer cells *in vivo*.

In their retrospective study, Huang et al. developed a radiomics-clinical predictive model for the response to neoadjuvant chemoimmunotherapy in patients with NSCLC. Their model integrates clinical and radiomic data from two institutions, drawing from a training and internal validation cohort of 105 patients and a second external validation cohort of 43 patients.

Sun et al. conducted an in-depth study on immune-related genes in hepatocellular carcinoma (HCC) using extensive datasets and robust bioinformatics methods, leading to the development of the Subtype-specific and Immune-Related Prognostic Signatures (SIR-PS) model. The SIR-PS model effectively predicted survival outcomes and immunotherapy responses in HCC patients, providing meaningful guidance for personalized immunotherapy.

High serum levels of hepatitis B surface antigen (HBsAg) increase the risk of developing HCC and have a worse prognosis for patients who have already developed HCC. Xiong et al. compared the effects of high and low levels of HBsAg in HCC patients undergoing transarterial chemoembolization (TACE) and sequential ablation and utilized propensity score matching to minimize selection bias. In addition, they created a nomogram to predict the prognosis of HCC patients with high levels of HBsAg after local treatment to more accurately guide the clinical decision.

Cancer incidence rises with aging, even though there are more senescent cells that have stopped dividing as we age. In their bioinformatics study, Ru et al. explored the molecular and immune landscape of cellular senescence in lung adenocarcinoma using publicly available TCGA and GEO datasets to gain deeper insights on the impact of cellular senescence on tumor progression. They showed that patients with low aging scores exhibited better survival, lower tumor mutation burden (TMB), lower somatic mutation frequency, lower tumor proliferation rate, and an immune-activated phenotype compared to patients with high aging scores.

Altogether, with this Research Topic, we primarily wanted to demonstrate that datasets from databases like TCGA and GEO, the former of which are being available and massively reanalyzed for more than a decade, are still relevant and useful for discovering new potential cancer drug targets, prognostic biomarkers, or therapeutic interventions, supported by new methods and ways of analyzing big data, especially now in the dawn of the development and application of AI models in basic cancer research. Even though such studies usually lack, at least *in vitro*, experimental validation, their results validated on external cohorts still present valuable and scientifically sound bases for further research and eventual translation into the clinical practice, while the amount of omics data continues to grow unstoppably…
